# ICoVax 2013: The 3^rd ^ISV Pre-conference Computational Vaccinology Workshop

**DOI:** 10.1186/1471-2105-15-S4-I1

**Published:** 2014-03-19

**Authors:** Anne S De Groot, Phoebe De Groot, Yongqun He

**Affiliations:** 1 EpiVax, Inc., Providence, RI, USA; and Institute for Immunology and Informatics, University of Rhode Island, Providence, RI, USA; 2 Unit for Laboratory Animal Medicine, Department of Microbiology and Immunology, and Center for Computational Medicine and Bioinformatics, University of Michigan Medical School, Ann Arbor, MI, USA

## Abstract

Following last year's computational vaccinology workshop in Shanghai, China, the third ISV Pre-conference Computational Vaccinology Workshop (ICoVax 2013) was held in Barcelona, Spain. ICoVax 2013 provided an international platform for the attendees to showcase their research and discuss problems and solutions in the development and application of computational vaccinology and vaccine informatics tools. The first of the three full-length papers presented at ICoVax discussed the discovery of viral "camouflage" through cross-conservation of T-cell epitopes using a tool called JanusMatrix. This important paper reports that viruses may camouflage their presence in the human body by incorporating sequences in their proteins that are highly cross-conserved at the T-cell receptor surface with human genome proteins, a discovery that has wide ranging implications for the development of vaccines against viruses that use the camouflage method. The other papers described a database for storing experimentally verified data on DNA vaccines and compared therapeutic targets of western drugs to Chinese herbal medicines for cardiovascular diseases. The short poster presentations covered various uses of informatics tools for processing the DNA and microRNA of pathogens to improve vaccine coverage, efficacy and development. A live (on-line) demonstration of the vaccine design toolkit, iVax, presented by Frances Terry of EpiVax, illustrated how computational vaccinology could be used in the design of next generation vaccines.

## Introduction and background

As a multidisciplinary field of research, computational vaccinology integrates computer science, mathematics, statistics, molecular biology, genomics, immunology and vaccinology. The capabilities of data processing and computational algorithms have dramatically increased in parallel with the number of genomes available for analysis. By leveraging the power of these data resources and algorithms, vaccine researchers are better positioned to design the next generation of vaccines, to study the fundamental immune responses that are relevant to vaccines, and to innovate in the field of vaccinology.

One means of advancing the field is to organize conferences where information on computational vaccinology approaches can be exchanged. The goal of the ISV Pre-conference Computational Vaccinology (ICoVax) workshops is to create an annual international forum for researchers to report, summarize, and discuss the most recent developments and ideas in the emerging areas of computational vaccinology and vaccine informatics. The most recent workshop took place immediately before the seventh Vaccine & ISV Annual Global Congress in Sitges, Spain on October 26, 2013. The first two ICoVax workshops took place in Seattle, USA and Shanghai, China in 2011 and 2012, respectively [1]. In total, three full-length papers, eight abstracts, and one software demonstration were presented at the meeting.. The three full-length papers were selected for extension and accepted for publication in a supplement in the journal *BMC Bioinformatics*.

## Summary of selected papers in the supplement

The three full-length original research papers accepted for the workshop were invited for extension and included in a special issue in the journal *BMC Bioinformatics*. These papers covered the use of informatics to reveal viral camouflage patterns by analyzing the cross-conservation of T-cell epitopes among different species [2], described a database for storing experimentally verified data on DNA vaccines [3], and compared therapeutics targets of western drugs to Chinese herbal medicines for cardiovascular diseases [4]. A brief introduction of these selected papers for the workshop is below:

The paper titled "Integrated assessment of predicted MHC binding and cross-conservation with self reveals patterns of viral camouflage" by He et al. introduces a new method for understanding T cell cross-reactivity between human-like and foreign protein sequences and how it can reveal patterns of camouflage in specific pathogens [2]. This method looks for sequence homology for only T Cell Receptor (TCR) facing ligands of the T-cell epitope that faces the TCR and generates the "Janus Immunogenicity Score" (JIS) based on how much cross-conservation and epitope has with the human genome and human microbiome [5]. The paper shows that putative T effector epitopes tend to have lower cross-conservation with self-epitopes, giving them high Janus Immunogenicity Scores. In contrast T regulatory epitopes had very high cross-reactivity with self and thus very low Janus Immunogenicity Scores. Furthermore, a pattern was observed in which viruses causing chronic infection (Epstein-Barr, HCV) had sequences skewed towards lower Janus Immunogenicity scores while viruses causing acute infection (Marburgh, SARS) had sequences skewed towards higher Janus Immunogenicity Scores. This tool is very promising for future research into the connection between virus immunogenicity, similarity with self and behavior and has already been incorporated into the vaccine design platform, iVAX.

The paper titled "DNAVaxDB: the first web-based DNA vaccine database and its data analysis" by Racz et al. introduces a new web-based DNA vaccine database DNAVaxDB (http://www.violinet.org/dnavaxdb) that stores over 400 manually annotated DNA vaccines for over 90 infectious and non-infectious diseases [3]. DNAVaxDB also includes the information of over 140 DNA vaccine plasmids and over 370 protective antigens used in DNA vaccine development. An analysis of these data resulted in the findings of specific patterns in areas such as subcellular localization of protective antigens and prime-boosting vaccination regimen. User-friendly web interfaces are available for the users to query the database information. This freely available database provides an easy platform to analyze the vast amount of information on DNA vaccines for future research in the field.

The paper titled "Target network differences between western drugs and Chinese herbal ingredients in treating cardiovascular disease" by Fu et al. presents a comparison of the target pathways of western drugs and Chinese herbal medicines using a database-derived network (4). The method analyzes western and Chinese herbal treatments for cardiovascular diseases, assessing the biochemical properties, regulated pathways and disease network. The findings show that Chinese herbal medicines tend to affect multiple signaling pathways and target the immune system, signal transduction and apoptosis associated pathways where as western drugs affected only a select few pathways and targeted upstream proteins regulating vascular smooth muscle contraction. The study also finds that the biochemical characteristics of Chinese herbal medicines may make them valuable sources for multi-target drug compounds or vaccine adjuvants.

## Summary of selected poster abstracts and software demo

Out of the eight poster abstracts accepted for the ICoVax 2013, seven abstracts were presented in short oral format and had associated posters exhibited at the workshop.

Five epitope-based abstract submissions were accepted in the workshop. The abstract "iVAX web-based vaccine design" [6] presented an integrated kit of web-based informatics tools that could be used to design epitope-driven vaccines in silico, directly from the pathogen genome sequence. "FastVax: Accelerated Vaccine Design, Production and Delivery for Emerging Infectious Diseases and Biodefense" [7] and "Broadly Reactive Influenza H1N1 CD4+ T-cell Epitopes Identified by Immunoinformatic Methods" [8] demonstrated how this platform could be used to quickly identify T-cell epitopes from pathogen genomes and develop highly-immunogenic, peptide-based vaccines in a short time frame, as is necessary in pandemic outbreaks. The FastVax program has been described in previously published articles [9]. The poster presentation "Low immunogenicity predicted for hemagglutinin of emerging avian-origin H7N9 influenza" [10] was followed up by an oral presentation at ISV (by author and ICoVax organizer, Annie De Groot). The presentation demonstrated how vaccine design tools could be used to analyze an emerging infectious disease pathogen's genome and predict the potential efficacy of the corresponding vaccine. The poster "Identification of T-cell Epitopes in the Hepatitis C Virus Genotype 4 Proteome: A Step towards Epitope-Driven Vaccine Development" [11] used the iVAX platform to identify HLA I and II T-cell epitopes that could confer protective immunity against HCV genotype 4, the most prevalent genotype in the Middle East and Central Africa, which has been largely ignored by past HCV vaccine development efforts.

Two gene- or MicroRNA-focused vaccine research posters were presented. The poster "A Pan-Genome Approach for Vaccine Design Against Johne's Disease in Dairy Herds" [12] showed how several live, attenuated vaccine candidates for Johne's disease were designed by using bioinformatics to conduct a large-scale genome analysis and identify target genes for developing attenuated mutant strains. "MicroRNA-vaccine relation inference through rule-based PubMed literature mining" [13] presented a tool for mining published literature on microRNA (miRNA) to help elucidate the relation between miRNA expression and the immune function of vaccines. Overall the posters demonstrated how informatics tools were effective in the processing gene sequences and expression for developing more effective vaccines.

Finally, the software demonstration of iVAX, the vaccine design and analysis tool used in several of presented papers, gave attendees the opportunity to see informatics-driven vaccine design in nearly real time. In addition to the related research that was presented, this demonstration presented the power and utility of immunoinformatics in the rapid development of next-generation vaccines [14].

## Competing interests

The authors were organizing committee members of ICoVax2013. Anne S. De Groot and Yongqun He served as co-chairs. ADG is the founder and majority shareholder at EpiVax, Inc., a privately owned vaccine design company located in Providence, Rhode Island, USA which developed and own the rights to the iVAX platform. ADG is also a faculty member at the University of Rhode Island Institute for Immunology and Informatics. This author acknowledges that there is a potential conflict of interest related to her relationship with EpiVax and attests that the work contained in this research report is free of any bias that might be associated with the commercial goals of the company. All other authors declare they have no other conflict of interest.

## Authors' contributions

ADG and YH were the lead editors for the workshop supplement. PDG contributed to the workshop organization, attended the workshop, recorded the proceedings and co-wrote the editorial. YH managed the Easychair paper and abstract submission system and organized the reviewing process. ADG chaired the workshop.
